# Individual Monitoring of Activity and Lameness in Conventional and Slower-Growing Breeds of Broiler Chickens Using Accelerometers

**DOI:** 10.3390/ani13091432

**Published:** 2023-04-22

**Authors:** Justine Pearce, Yu-Mei Chang, Siobhan Abeyesinghe

**Affiliations:** The Royal Veterinary College, Hawkshead Lane, North Mymms, Hatfield AL9 7TA, UK

**Keywords:** animal welfare, broiler chicken, behavior, activity, accelerometer, leg health, poultry, walking ability, bio-logging

## Abstract

**Simple Summary:**

Broiler chickens are specifically bred for meat, and conventional breeds have been genetically selected for faster growth rates. Rapid growth is associated with a greater risk of lameness in broiler chickens, which is a major welfare concern associated with an economic loss for producers. Although defined as an individual’s physical and mental state, welfare is often monitored at the group level and is at risk of overlooking individual issues. Body-mounted accelerometers measure movement and could potentially monitor individual welfare, including lameness. However, body-mounted sensors may alter behavior and/or impact welfare. We investigated relationships between accelerometer-recorded activity (all movement), weight, and lameness in broiler breeds with different growth rates. Wearing the device did not negatively impact lameness, and, except for preening more, birds behaved similarly to those not wearing accelerometers after 24 h. We found that faster-growing, heavier birds were less active compared to slower-growing, lighter birds, demonstrating that accelerometers could detect known influences on activity useful for general monitoring, but that accelerometer-detected activity was not linearly associated with lameness. Further research on accelerometer detection of more specific behavioral changes likely to be associated with lameness, such as non-linear acceleration with gait score, offers the possibility of better continuous monitoring of individuals to assess lameness objectively.

**Abstract:**

Accelerometers are increasingly being investigated to detect animal behavior as a method for monitoring individual welfare that overcomes manual challenges associated with time, resource, and discrete sampling. We investigated the effects of broiler chicken hybrid (hereafter breed) and weight on accelerometer activity (activityA; calculated as percentage of time spent active (%)) and its association with lameness as a major broiler welfare concern. Accelerometers were attached to birds of different breeds on between 2 and 4 occasions from 26 to 30 days old (conventional breed CNV) and 26 to 49 days old (two slower-growing breeds SGH; SGN). At 2.2 kg, lameness was scored using a 6-point gait scoring system (0: unaffected to 5: severely lame). Linear mixed effects models and breed-stratified generalized linear models together with a random-effect meta-analysis were used for data analyses. ActivityA was lower in faster-growing, heavier birds compared to slower-growing, lighter birds, showing overall consistency with previous behavioral research, but did not vary linearly with gait score. Accelerometers offer the potential for simple broad-scale continuous monitoring of broiler chicken activity behavior that requires limited data processing. Exploration of the ability of accelerometers to capture more subtle and specific changes in behavioral patterning, such as non-linear acceleration with gait score that could indicate early development of lameness, warrants further investigation.

## 1. Introduction

Conventional broiler chickens have been genetically selected for rapid growth and larger breast muscles, resulting in heavier, morphologically abnormal birds [1]. Due to developmental abnormalities, as well as some degenerative and infectious disorders [2,3], broiler chickens commonly experience poor leg health, including lameness [4]. Poor leg health and skeletal problems can result in culling, mortality, reduced feed efficiency, and growth, and these issues, along with treatment and prevention costs, are said to be the main cause of economic losses in broiler flocks [3,5,6]. Lameness (defined as an obvious abnormality to an incapable ability to walk) was recently recorded as prevalent in 53% of broiler chickens, with an additional 44% of birds demonstrating slight abnormalities [7], and it is considered one of the most severe welfare problems seen on modern farms [8]. Manual on-farm assessment of poultry lameness is challenging, time consuming, expensive, may disturb or distress birds, and is usually undertaken at a single cross-sectional sample point toward the end of production [9,10]. This approach cannot represent lifetime welfare and is retrospective, disallowing intervention and management within the production cycle. Early intervention could be possible with wearable sensors able to detect behavioral changes associated with lameness [8,11].

Increasing research into precision livestock farming (PLF) has opened up new potential for real-time, automatic monitoring of animal welfare, encompassing behavior and health (e.g., the eYeNamic™ image processing system to monitor flock distribution and activity [12]; the optical flow technique that monitors overall flock activity to identify changes in behavior and has shown successful correlations with gait score (suggesting lameness [13]), hock burn, and footpad dermatitis [14]). However, the majority of these approaches have remained focused on group-level outcomes. Most definitions of animal welfare focus on the individual’s physical and mental state, dependent on the individual’s unique experiences, genetic makeup, and preferences [15,16]. Thus, it can be argued that welfare should be assessed at the individual level. Wearable biosensors, such as accelerometers, allow for this individual approach and continuous focal sampling [17,18,19].

Body-mounted sensors to record locomotor dynamics, such as accelerometers, are becoming increasingly used to record the activity of livestock as an alternative method for monitoring welfare and disease that is time-saving, continuous, and objective [16,17]. Lame birds spend less time walking and more time sitting compared to sound birds [8,11]. Breed differences are also seen. When reared in similar environments, faster-growing genotypes are more susceptible to leg health problems [5,11,20] and show more inactive sitting and side-lying and less standing and walking compared to slower-growing genotypes [11,21]. Accelerometer activity thus offers the potential to capture changes in behavior that occur with lameness.

Activity is a term used variably within the literature, but in general, it is used to mean any bodily movement caused by skeletal muscle [22]. Overall dynamic body acceleration (ODBA) and vectorial dynamic body acceleration (VeDBA) are two features used to incorporate acceleration information from all three accelerometer axes, and both offer useful and simple methods for using accelerometry data [23]. ODBA is the sum of all three axes’ dynamic acceleration without static acceleration squared, and VeDBA is calculated as the square root of ODBA [23,24]. Halsey et al. [25] successfully used accelerometer activity, measured using ODBA, to estimate the energy expenditure of laying hens. Similarly, Okada et al. [26] were able to detect abnormal states in chickens caused by avian influenza using VeDBA. However, there are limited data investigating the use of accelerometers to measure activity in broiler chickens specifically, and although body-mounted sensors are not invasive [18], they are potentially intrusive and can result in changes in behavior or affect welfare [19] due to both impacts of sensor weight and position as well as changes in appearance. Dennis et al. [27] found that changing appearance with livestock markers on the tails of birds had no effect on aggression or feather pecking compared with unmarked controls. Conversely, Rault and Taylor [28] found that chickens showed increased fear toward birds marked with blue (at the tips of their wings and on their rump), but this fearful reaction decreased 1 day after spray marking. It is, therefore, necessary that when using any methods that alter the appearance, potential behavioral effects are monitored, and an appropriate habituation period is scientifically justified.

When attaching accelerometers to birds, various methods have been explored, including backpack attachments whereby a device is placed into a casing with straps looping around the birds’ wings (laying hens: [29,30,31]; broilers: [32,33]), as well as Hypafix^®^ tape methods, whereby a device is fitted directly to the bird’s skin (guinea fowl: [34]). Although backpacks have been previously successful, they are at risk of acceleration signal disturbance as the sensors can move independently from the bird’s body and are not in a fixed position. This can negatively affect the signal quality when collecting acceleration data. To overcome this problem, Hall et al. [34] used tape to attach accelerometers to the backs of guineafowl, which resulted in improved sensor stability and caused minimal behavioral disturbances. Buijs et al. [30] found that laying-hen behavior was affected immediately after backpack attachments (birds with backpacks performed significantly more sitting/lying, preening, side-stepping/reversing, and pecking the equipment and performed significantly less standing and walking compared to conspecifics with no backpack) but, with the exception of self-pecking the equipment, these differences subsided after 2 days, suggesting habituation to the attachment after this time. Additionally, Buijs et al. [30] found no effect of backpacks on bird body weight gain. Similarly, Stadig et al. [32] found that neither growth, hock burn, footpad dermatitis, cleanliness, nor gait differed between birds with and without backpacks, suggesting that backpacks had no negative consequence on a slower-growing hybrid (hereafter referred to as breeds) of broiler welfare between a 35–70-day-old period. However, since weight is known to be associated with an increased risk of lameness in conventional broiler chickens [35,36], it is possible that carrying even a slight additional weight might additionally compromise locomotion and exacerbate lameness as well as differences in lameness between breeds. In broilers, Dawson et al. [33] successfully used accelerometers to monitor differences in breed inactivity (0 acceleration signals) using the ‘backpack’ method but did not investigate any effects of accelerometer attachment on bird behavior or welfare outcomes. Crucially, the literature on attaching sensors to broilers is limited, and both short-term responses and longer-term effects require further research. Similarly, it is important to ensure that accelerometer data used to monitor welfare are not an artifact of wearing a device. To our knowledge, no other studies have investigated the effects of accelerometer attachment on broilers using the tape method associated with improved sensor stability.

The use of PLF technology for poultry farming remains limited as some PLF systems require further validation prior to commercial use (e.g., the eYeNamic™ cameras are commercially available, but the monitoring systems themselves were categorized as prototypes [37]). Real-time monitoring of large data sets can be costly, and the payback period for investment is uncertain [38]. The use of accelerometers on farms is currently impractical, particularly if devices were to be attached to multiple birds. However, with advances in technology, reductions in size, and appropriate attachment methods that do not interfere with the food chain, accelerometers could potentially offer information on focal or sentinel birds within a flock, as our previous work demonstrated that behavior of 10% of a group represents the pen prevalence of health outcomes [11]. Moreover, in the interim, accelerometers may prove advantageous tools for the selection of valuable breeding birds, allowing the capture of key behavior traits that are otherwise challenging to measure consistently. Additionally, the use of accelerometers could benefit researchers or veterinarians investigating lameness as automated techniques reduce the need for human-based gait scoring systems. However, more conclusive evidence on the association of accelerometer data with welfare outcomes is required as proof of principle. We aimed to investigate: (1) the effects of breed, sex, and weight on accelerometer activity (activityA) measured using VeDBA and (2) the association between activityA and weight on gait score. It was hypothesized that slower-growing breeds would show higher activityA levels compared to the faster-growing breed, that, across breeds, the activityA levels would decrease with weight, and that birds with higher gait scores (worse lameness) would show reduced activityA. The secondary aims were to investigate the effects of accelerometer attachment on bird behavior immediately and at 24 h post attachment as well as impacts on welfare outcomes at the end of production.

## 2. Materials and Methods

The work was approved by the Royal Veterinary College Clinical Research Ethical Review Board (reference: URN 2018 1814-3, received on 26 June 2018).

### 2.1. Subjects, Housing, and Husbandry

Subjects comprised an opportunistic sub-sample of birds (*n* = 55) from a wider study described in Abeyesinghe et al. [11]. The latter focused on examining associations between health outcomes and behavior across different breeds reared under a modification of the Royal Society for the Prevention of Cruelty to Animals Broiler Breed Welfare Assessment Protocol (RSPCA BBWAP) [39]. Abeyesinghe et al. [11] incubated and hatched fertilized eggs from one faster-growing, conventional breed (CNV) and two slower-growing breeds (SGH and SGN), and randomly allocated chicks (*n* = 150 CNV, 250 SGH, 150 SGN) to same-breed pens (6.5 m^2^, to achieve a stocking density of 7.7 birds/m^2^ up to 2.4 kg (18.5 kg/m^2^) according to the RSPCA BBWAP [39]) of 50 birds within identical environment-controlled rooms. There were three pens within each room, and, where possible (hatch rate dependent), each room consisted of one pen of each breed randomly assigned to pen position (front, middle, or back of room). Pens were bedded with wood-shavings, a bell drinker and bell feeder (22 mm and 150 mm trough space per bird, respectively) and a 1.3 m length wooden perch (positioned at a height to allow birds easy access dependent on the age and breed of the bird, typically between 10 and 30 cm as per the RSPCA BBWAP [39]). Non-limiting feed was provided according to the RSPCA BBWAP [39]. Birds were reared with a 22 h light:2 h dark schedule (daylight strip bulbs, dawn onset at 05:30) to three days, then the dark period increased by 1 h per day to a maximum of 18 h Light:6 h Dark. On day 5, five birds per pen (*n* = 15 CNV, 25 SGH and 15 SGN) were randomly selected as subject focal birds for the current study and marked using non-toxic spray and leg tags, which were checked and replaced weekly.

### 2.2. Accelerometer Attachment

**Figure 1:**
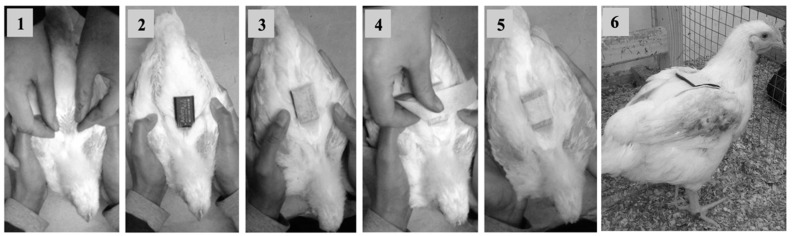
Images showing the process of attaching the accelerometers using tape. (**1**) Part the feathers to reveal skin; (**2**) fix accelerometer to skin using double-sided tape; (**3**) place Hypafix^®^ adhesive tape vertically over the top of the accelerometer (parting feathers as much as possible); (**4**) place Hypafix^®^ adhesive tape horizontally over the top of the accelerometer and underneath wings; (**5**) complete tape attachment; (**6**) complete attachment with vest used to protect the tape attachment from external elements (e.g., dust) and to keep the accelerometer secure.

Accelerometers (AX3 Axivity logger (Axivity Ltd., Newcastle, U.K.), 23 × 32.5 × 7.6 mm and 11 g) were attached to focal birds on between 2 and 4 occasions for a maximum period of 32 h on each occasion until they reached an average slaughter live weight of 2.2 kg. Accelerometers were attached between 08:00 and 11:59 am on day 1 to allow a minimum of 24 h habituation prior to accelerometer data recording on day 2. At the time of each accelerometer attachment, bird weight (kg) was recorded. Due to breeds reaching slaughter weight at different times, accelerometers were attached to birds from 26 to 30 days old for the CNV and 26 to 49 days old for the SGH and SGN breeds (Table 1). Birds were removed from the home pen to attach accelerometers and placed back into their home pens within 10 min after attachment. Feathers in the center of the birds’ backs were parted to reveal skin to which the device was secured using double-sided tape and 2 pieces of 5 cm Hypafix^®^ adhesive tape over the top (Figure 1) (as used previously for guinea fowl [40]), which removed few to no downy feathers upon later removal. Vests comprising Lycra stretch fabric then covered the accelerometer, passing longitudinally under the bird’s abdomen between the legs and over the back, with holes cut for the head and tail. Three vest sizes were cut to suit birds at different ages and were more specifically adjusted to fit individuals at time of attachment, when birds were additionally weighed.

The accelerometer configuration settings were set to 100 Hz logging and range ±8 g (here units of ‘g’ represent the magnitude of acceleration due to gravity). The open-source OMGUI Configuration and Analysis Tool software (Newcastle University, Newcastle, U.K., 2015) was used to set up and configure the accelerometers to record as well as to download and visualize the recorded data from individual sensors.

### 2.3. Effects of Automated Monitoring Equipment on Behavioral Habituation

To investigate behavioral habituation to accelerometer wearing, 30 accelerometer birds were observed (H-Accel) (*n* = 10/breed; 2 pens/breed) and 30 control birds (unmarked birds from the same pens not wearing accelerometers (H-Con)) were observed immediately, half an hour, 1 h, and 24 h post first attachment (26 days of age). The frequency of behavior (Table 2) was recorded for 5 min at each sample point using continuous focal sampling. H-Con birds were randomly selected and observed at each time point to act as a control for factors such as diurnal variation.

### 2.4. Welfare Assessment

As part of the wider study [11], once the overall weight of each breed averaged a live weight of 2.2 kg (measured by bulk weighs), all birds of that breed were weighed (final weight) and welfare assessed, which included sexing and gait scoring [11,39]. Birds reached this average live weight at 34 (CNV), 46 (SGH), and 53 (SGN) days old, respectively, and it was on this day that each breed was welfare assessed. Gait score was recorded using the BBWAP 6-point Gait Scoring System [39] modified from Kestin et al. [43] (Table 3). Each bird was gently placed at the start of a meshed corridor within the pens and observed for at least 10 steps while they moved down the corridor to re-join the other birds. Two observers scored independently and then agreed a score for each bird’s gait. Assessments were carried out by the same individuals each time.

### 2.5. Effects of Wearing an Accelerometer on Weight and Gait Score

To investigate any effects of automated monitoring equipment on bird weight and gait score, final weight and gait score data from three groups of birds were collated: (i) birds with accelerometers (W-Accel; *n* = 5 per pen), (ii) spray-marked birds (W-Spray; *n* = 5 per pen): birds that were handled and spray-identified the same as accelerometers birds to account for the effects of repeated handling, (iii) control birds (W-Con; *n* = 5 per pen): birds from the same pen that were un-handled (with the exception of bulk weights at 1, 2, 4, and 5 weeks of age and the welfare assessment), and not identified with any spray marking. Data from W-Spray birds and W-Con birds were collated as part of the wider RSPCA BBWAP study [11] and used here for comparison. Each group consisted of 15 CNV, 25 SGH, and 15 SGN (total *n* = 165).

### 2.6. Accelerometer Data Processing for Activity

Using MATLAB (version 9.9.0.157001, R2020b), activityA was calculated across a 4 h time segment from each individual bird’s accelerometer trace between 10.15 and 16.15 on day 2 of accelerometer attachment. To reduce noise (here referring to acceleration signal disturbance) [44], acceleration data were filtered using a Butterworth bandpass smoothing filter with cut-off frequencies of 1.79 Hz and 21.70 Hz to remove frequencies below and above the frequency range of behaviors in broilers. The data were then further smoothed by calculating the birds’ vectorial dynamic body acceleration (VeDBA; Equation (1)) [24] as the single, integrated measure of body motion because of its lower error rate when monitoring low activity, likely to be shown by broilers, compared to ODBA [45].

VeDBA = √X^2^ + Y^2^ + Z^2^ (1)

When calculating VeDBA, data are commonly summarized over periods of time known as window lengths or epochs. To calculate activityA, VeDBA was smoothed across a 1 s window (VeDBA_1 s), and a VeDBA activity threshold of 0.05 g was used, i.e., when a bird’s VeDBA was above 0.05 g, the bird was deemed active and, below 0.05 g, inactive. This activity threshold was selected on the basis that when an animal is resting, its activity level is likely to be close to zero, while more energetic behaviors result in higher levels of activity [46]. The total percentage of time spent active (activityA) was subsequently calculated using Equation (2):

Total % of Time Spent Active = (Sum of total number of labels classed as active measures/total length of VeDBA_1 s smoothed dataset) × 100.(2)

### 2.7. Statistical Analysis

Statistical analysis was carried out using SPSS (IBM SPSS Statistics, version 27) and R (version 4.1.0). 

#### 2.7.1. Evaluating the Effects of Wearing an Accelerometer on Behavior

To investigate the effect of accelerometer attachment on behavior across a 24 h period, a generalized estimating equations model with Poisson loglinear link function was employed. An exchangeable working correlation matrix structure was used to account for repeated individual bird measures across time. The model included group effect (H-Accel vs. H-Con), time effect (immediately, half an hour, 1 h, 24 h post attachment) and group by time interaction effect. If the interaction effect was significant, post hoc comparison was performed to identify any group differences at specific time points [47]. Estimated effect was presented as rate ratio (RR). 

#### 2.7.2. Evaluating the Effects of Wearing an Accelerometer on Weight and Gait Score

To investigate any effects of automated monitoring equipment on bird weight (final weight) and gait score, a linear model and a generalized linear model with ordinal cumulative logit link function were used to respectively evaluate the effect of accelerometer attachment on final weight (kg) and on gait score. The latter model included the effect of breed and its interaction with a group (W-Accel, W-Spray, and W-Con) as the gait score was likely to be partially confounded with the breed. Mean ± standard deviation was presented for descriptive statistics.

#### 2.7.3. Evaluating the Effect of Breed, Sex, and Weight on ActivityA

To evaluate the effects of breed, sex, and repeated weight (weight recorded at the time of each accelerometer attachment) on activityA, a linear mixed effects model was used. The random effect of the bird was included in the model to account for repeated measures from the same bird. The residual plot was visually inspected to ensure the assumption of normal distribution was met. Mean ± standard deviation was presented for descriptive statistics.

#### 2.7.4. Evaluating the Association between ActivityA and Gait Score

To evaluate the association between activityA and repeated weight on gait score, a breed-stratified generalized linear model together with random-effect meta-analysis was used. A generalized linear model with an ordinal cumulative logit function was run for each breed separately. As the outcome gait score was taken as a single measure toward the end of the bird’s life, the mean activityA and mean repeated weight recorded in the week prior to the gait score measurement were used for these analyses (activityA and repeated weight measures taken earlier were excluded). Results were presented as odds ratio (OR) and 95% confidence interval (CI). Subsequently, the estimated OR and CI of each individual breed were summarized using a random-effect meta-analysis model. Heterogeneity between breeds was evaluated using I^2^ statistics. This was an attempt to better account for the breed as a potential confounder in the analysis and to evaluate the usefulness of activityA as a sole predictor of gait. The aim was to explore the dispersion of effect sizes for each breed between activityA and gait score.

## 3. Results

### 3.1. The Effect of Accelerometer Attachment on Behavior

**Figure 2:**
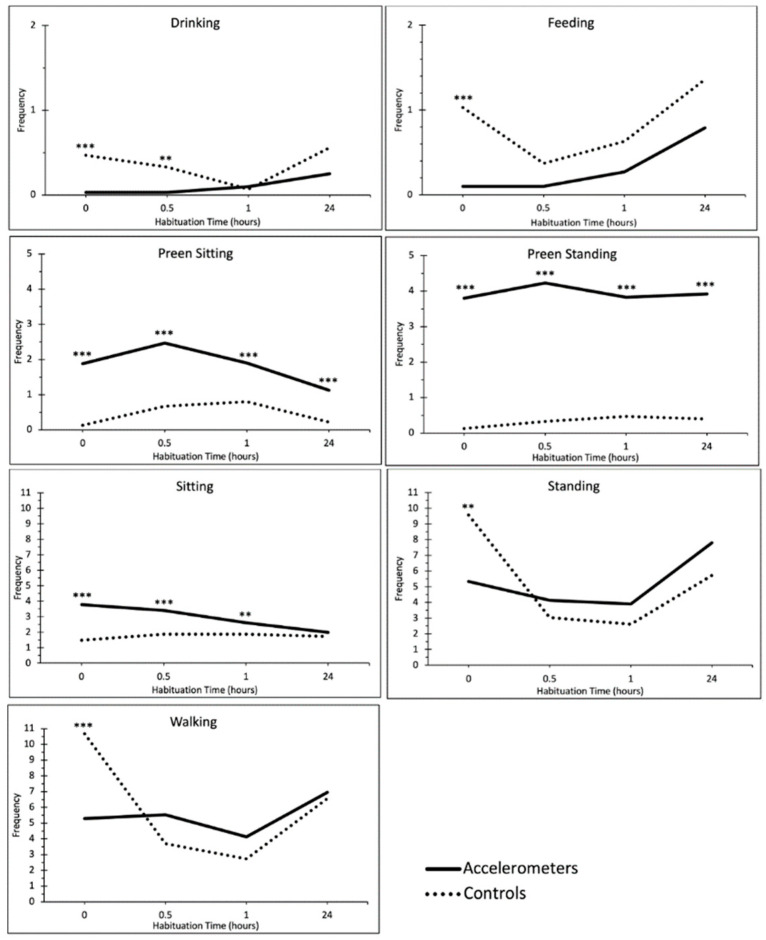
Line graphs showing the mean frequency for each behavior; visualizing the interaction effect of behavior frequency (recorded within a 5 min continuous, focal observation period) over time between accelerometer and control birds. *p* values: ** *p* < 0.01, *** *p* < 0.001.

Figure 2 shows the changes in behavior frequency between the accelerometer and control birds over time following the return to the home pen after the accelerometer attachment. 

Birds wearing accelerometers showed more frequent preen standing behavior overall compared to control birds and this persisted across the 24 h (group × time interaction = X^2^(3) 3.71, *p* = 0.295; group effect = X^2^(1) 115.36, *p* < 0.001; time effect = X^2^(3) 3.48, *p* = 0.323) (Figure 2).

There were significant interaction effects between the group and time for drink (*p* = 0.007), feed (*p* = 0.001), preen sit (*p* = 0.017), sit inactive (*p* = 0.001), stand inactive (*p* = 0.001), and walking behavior (*p* < 0.001). H-Accel birds drank less frequently than H-Con birds immediately after attachment (RR = 0.07, *p* = 0.009), but this difference subsided by 1 h (RR = 1.50, *p* = 0.683). H-Accel birds fed less frequently than H-Con birds immediately after attachment (RR = 0.10, *p* < 0.001), but this difference subsided by half an hour (RR = 0.27, *p* = 0.068). H-Accel birds sat preening more frequently than H-Con birds immediately after attachment (RR = 14.13, *p* < 0.001), and this difference remained significant at 24 h (RR = 5.1, *p* = 0.003). H-Accel birds sat inactive more frequently than H-Con birds immediately after attachment (RR = 2.57, *p* < 0.001), but this difference subsided by 24 h (RR = 1.15, *p* = 0.364). H-Accel birds stood inactive less frequently than H-Con birds immediately after attachment (RR = 0.56, *p* = 0.014), but this difference subsided by half an hour (RR = 1.36, *p* = 0.275). H-Accel birds walked less frequently than H-Con birds immediately after attachment (RR = 0.50, *p* < 0.001), but this difference subsided by half an hour (RR = 1.50, *p* = 0.165) (Figure 2).

### 3.2. The Effect of Accelerometer Attachment on Weight and on Gait Score

There were no significant differences in final weight between birds fitted with an accelerometer, spray birds, and control birds (group) (X^2^(2) 0.04, *p* = 0.979), and although there was a significant breed effect (final weight: CNV = 2.2 ± 0.28 kg, SGH = 2.8 ± 0.26 kg, SGN = 2.4 ± 0.28 kg; X^2^(2) 44.13, *p* < 0.001), the interaction between breed and group was non-significant (X^2^(4) 6.99, *p* = 0.137).

There was a significant group effect on gait (X^2^(2) 7.04, *p* = 0.03) with W-Accel birds at increased odds of scoring lower gait scores than W-Spray (OR = 2.39, *p* = 0.03) and W-Con (OR = 2.75, *p* = 0.01). There was also a significant effect of breed on gait (gait score: CNV = 2.32 ± 0.64, SGH = 1.43 ± 0.50, SGN = 1.22 ± 0.67; X^2^(2) 44.13, *p* < 0.001), but the interaction between group and breed was non-significant (X^2^(4) 2.25, *p* = 0.691).

### 3.3. Evaluating the Effect of Breed, Sex, and Weight on ActivityA

Sex ratios by breed were as follows (F:M): CNV 9:6, SGH 14:11, SGN 8:7; Total 31:24 (Table S1: Descriptive statistics demonstrating data distribution for each breed). There was no effect of sex on activityA (sex: F(1) 0.560, *p* = 0.458), and sex was subsequently removed from the model. Effects of breed (F(2) 20.515, *p* < 0.001) and repeated weight (F(1) 96.524, *p* < 0.001) both remained significant. SGN performed significantly higher activityA levels (26.57 ± 5.83%) than SGH (21.85 ± 6.12%, *p* = 0.001), and these two breeds respectively showed significantly higher activityA than the CNV breed (17.61 ± 3.32%, *p* < 0.001) (Table S1: Descriptive statistics demonstrating data distribution for each breed). ActivityA reduced by 6.6% per 1 kg increase in repeated weight (F(1) 96.524, *p* < 0.001). A further model was run to test for any interaction between repeated weight and breed, which showed no significant interaction (F(2) 0.457, *p* = 0.635).

### 3.4. Evaluating the Association between AcitivityA and Gait Score

Overall activityA across breeds decreased linearly from gait score 0–2 (Table S2: Descriptive statistics of last week mean activityA (%) and last week mean weight (kg) for each gait score and breed). Breed and gait score were correlated whereby slower-growing breeds represented the lowest scores and faster-growing breeds highest scores; the overall range was limited to scores 0–3 (gait score range by breed: CNV 1–3; SGH: 1–2; SGN: 0–2; Table S1: Descriptive statistics demonstrating data distribution for each breed). 

**Figure 3:**
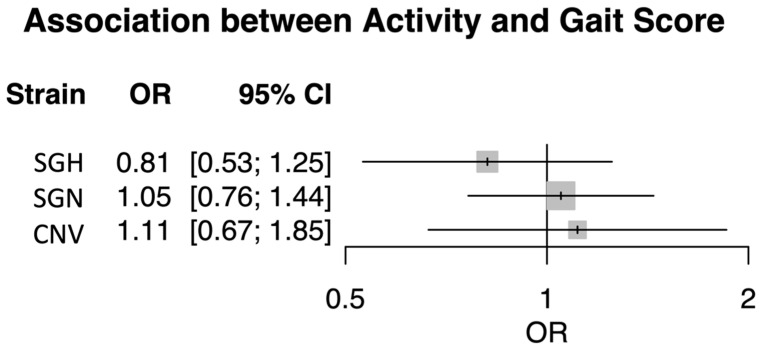
This forest plot shows that the three broiler breeds did not demonstrate significantly different relationships between activityA and gait score (estimated heterogeneity of variance: 0, *p* = 0.573). The summary effect is close to 1, and a very narrow confidence interval covering one suggests no overall significant relationship between activityA and gait score (summary effect = 0.99, CI [0.79, 1.24]).

The generalized linear model results used for the meta-analysis can be found in Table S3: Generalized linear model results summarizing the relationship between gait score and the mean activity^A^ (%) and mean repeated weight (g). The confidence interval of the pooled OR from the meta-analysis covered one, showing a non-significant relationship between gait score and activityA across the three breeds (pooled OR = 0.99, CI [0.79, 1.24]). No heterogeneity was detected, and the variation between the three breeds was found to be non-significant (estimated heterogeneity of variance: 0, *p* = 0.573) (Figure 3).

## 4. Discussion

We aimed to investigate (1) the effects of breed, sex, and weight on accelerometer activity (activityA) and (2) the association between activityA and weight on gait score. We also took the opportunity to investigate the effects of accelerometer attachment on bird behavior immediately and at 24 h post attachment as well as impacts on welfare outcomes at the end of production. As expected [30], behaviors differed substantially between birds with and without accelerometers immediately after attachment, but by 24 h habituation, the effects of device attachment were visible in only preening behavior. Buijs et al. [30] also found that the attachment of backpacks resulted in accelerometer birds preening significantly more immediately after attachment compared to control birds, but this difference subsided after 2 days. Further work is needed to establish whether differences in preening seen with the tape attachment also subside after 24 h, or with repeated attachment, or whether this represents a persistent disruption in behavior requiring further exploration of accelerometer attachment. However, no differences in the final weight, suggestive of impacts on growth, were found between birds with and without accelerometers. Furthermore, although there was a significant group effect on gait score, birds with accelerometers were at increased odds of scoring lower gait scores, suggesting a lower risk of lameness and no longer-term impacts of accelerometer wearing on welfare outcomes. Although it is not currently possible to determine why the gait scores of birds wearing accelerometers might have been lower, the differences observed were in the mild gait score range (below gait score 2). It is unlikely that repeated wearing of accelerometers for short time periods from 3 weeks of age onward positively impacted musculoskeletal strength and this could be an artifact of the random selection of birds. However, the main point of note is that accelerometer wearing did not compromise welfare by increasing the risk of lameness. Consequently, the method of attachment using tape on young broilers was deemed successful, requiring a short habituation period following attachment and prior to data collection.

Our findings strongly supported the predicted effect of breed and weight on activityA and, as expected, slower-growing and lighter birds demonstrated higher activityA levels compared to faster-growing and heavier birds. These findings correspond with previous behavioral work where faster-growing breeds showed more inactive behaviors (sitting and side-lying) and less active behaviors (walking) compared to slower-growing breeds [11,21], and birds with lighter body weights showed more active behavior [48]. Therefore, accelerometers (and VeDBA) can discriminate the effects of key known broiler characteristics on activity and have the potential to capture bird behavior.

As expected [37], faster-growing birds scored higher gait scores compared to the slower-growing breeds, suggesting that lameness was less prominent in slower-growing birds at similar weights. However, in contrast to previous findings [49,50,51], a negative association between gait score and activityA was not supported. There are a number of possible explanations for this finding. Aydin et al. [49] suggest that differences in activity (measured using EYeNamic™ software) compared to non-lame birds may be more distinguishable in clinically lame birds with gait scores of 4 or 5, which were not observed in our study as birds of a gait score of 4 or more were immediately culled on welfare grounds. Aydin et al. [49] divided 30 birds (Ross 308) selected from a commercial farm based on their gait score (5 birds per gait score 0–5). This suggests that we would need more birds per breed showing a gait score range of 0–5 to better understand the potential activityA offers in capturing changes in behavior that occur with lameness. Aydin et al. [49] also found that the relationship between gait score and accelerometer-measured activity was not linear, with activity reducing between gait scores 0, 1, and 2 but counter-intuitively increasing in birds with gait score 3. Although not formally tested, we observed this non-linear pattern of reduction in activityA between gait scores 0 and 2, with a slight increase at gait score 3 for the overall activityA across breeds and within the CNV and SGH birds. Accelerometer activity will detect movement indiscriminately, including normal behavior as well as disturbances and abnormal movement. Thus, for higher gait scores, the alterations in movement (e.g., greater accelerations) with uneven gait patterns may be detected as ‘more’ active, as might small movements or more frequent positional changes to ease discomfort associated with lameness during rest, potentially explaining this increased accelerometer activity. If a non-linear relationship was consistent, a non-linear algorithm to detect gait score using consistent trends in activity would be worth exploring. However, in SGN birds, both activityA and weight increased linearly with gait score, suggesting breed differences in activity patterns associated with lameness. Differences in activity observed may be explained by different causes of lameness. Physical abnormalities such as tendon degeneration or rotated tibia associated with faster growth rates are conditions that may cause pain [21]. However, it is also possible that not all lameness conditions are painful, and some may be a result of biomechanical differences between breeds. An increase in activityA at a gait score of 3 could be an artifact of disturbed and uneven movements associated with larger bird morphologies [52,53]. There is evidence that morphological characteristics, as a result of selective breeding for larger pectoral muscle mass and increased meat yield, can result in differences in gait [52]. Therefore, it is possible that a heavy bird with a high gait score may move more exaggeratedly compared to a smaller bird with the same or a higher gait score. This would result in differences in accelerometer-recorded acceleration in different axes, which are combined with the overall activity measure. Additionally, the SGN had undergone much less selective breeding and could therefore have been potentially less morphologically impacted by the selection, explaining the different apparent pattern. However, lame birds could alternatively perform similar total durations of some behaviors as sound birds but with altered temporal and/or spatial patterning. For example, Weeks et al. [8] report lame birds fed less frequently and for longer durations per bout than sound birds but with no overall difference in the total duration of feeding behavior per day. As the total percentage of time spent active is most likely to correspond with total durations of a range of behaviors, it is unlikely activityA would pick up on differences in the temporal patterning of behavior within general activity, potentially explaining why we observed no differences in activityA between lame and sound birds. Nonetheless, while it was not possible to predict the final gait scores of our accelerometer birds, the restricted within-breed variation in gait score and the sample size is likely to have impacted our study. Further research addressing these issues is warranted to unpick these alternative explanations and establish if breed-specific interpretations of activity data as well as gait-score-specific accelerometer characteristics are necessary to establish this measure or if accelerometer measurement of more specific behaviors offers more scope for monitoring broiler lameness. As accelerometers are able to monitor specific movement characteristics measured along the *X*, *Y,* and *Z* axes [24,29,54,55], it is possible that alternative accelerometer parameters specific to each axis may be more successful at identifying subtle changes in gait suggesting lameness. These parameters would require further investigation, specifically using more computationally advanced processes such as machine learning algorithms.

## 5. Conclusions

In line with previous findings on the effect of breed and weight differences on activity [11,21,33,48], the hypothesis that breed and weight would have an effect on activityA was strongly supported by this research. This suggests that accelerometers have the potential to capture bird behavior, and activityA offers a simple, broad-scale method for continuous monitoring of focal individuals that requires minimal data processing. The hypothesis that there was a negative association between activityA and lameness was not supported by this study’s findings. Although previous work has suggested a non-linear relationship between activity and lameness [49], our findings suggest there may be breed differences in activityA patterns associated with lameness. Morphological breed differences known to impact gait [52] may explain these apparent breed differences in activityA observed. Further research working toward behavioral validation of accelerometer activity would be important to ensure devices are recording what is expected and any detection of abnormalities is robustly tested and validated across different breeds. Furthermore, it is possible that, rather than overall activity, specific behaviors measured by accelerometers could indicate early development of lameness and afford earlier intervention.

## Figures and Tables

**Table 1:** Method timeline demonstrating when accelerometers were attached to and when welfare assessments were carried out on the different breeds and at what age.

Age (Days)	26	27	28	29	30	31	32	33	34	35	36	37	38	39	40	41	42	43	44	45	46	47	48	49	50	51	52	53
CNV (N = 15)	A	D			A	D			WA																			
SGH (N = 25)	A	D			A	D									A	D					WA		A	D ^1^				
SGN (N = 15)	A	D			A	D									A	D							A	D				WA

A = Accelerometer attached, weight measured (kg), Acclimatisation; D = Data recorded for focal birds, accelerometers removed at end of data recording; WA = 2.2 kg strain weight met, Welfare assessment performed (gait score, sex recorded); 1The data collected for SGH birds on day 49 was opportunistic. It was assumed that the lameness score assessed on day 46 would still apply on day 49.

**Table 2:** Ethogram for continuous focal observations of broiler behaviors immediately, half an hour, 1 h, and 24 h post accelerometer attachment.

Behaviors	Definition (Modified from An Ethogram Supplied by University of Guelph as Part of An Alignment of Methods in the Wider Study [11])
State Behaviors (Mutually Exclusive) Measured as Frequencies	
Walking	Slow movement forward where one foot is always placed on the ground and breast is above ground. Start from movement, a slight shift in body weight just before foot is raised off ground. Ends when both feet are placed onto the ground and when neither foot has moved for 2 s or, when another behavior commences.
Sit Inactive	Sat down, immobile with entire breast touching the ground and legs tucked underneath bird. Start from cessation of movement for 2 s. Ends when another state behavior commences (event behaviors can occur simultaneously).
Standing	Immobile on both legs with body not touching ground. Start from cessation of movement for 2 s. Ends when another state behavior commences (event behaviors can occur simultaneously) (modified from [41]).
Feeding	Downward pecking in feeder while sitting or standing. Start from the first peck at feed. Ends when bird has not pecked at feed for 3 s or when another behavior commences (modified from [42]).
Drinking	Downward pecking in drinker while sitting or standing. Start from the first peck in drinker defined as direct beak contact with water. Ends when bird has not lowered head to drink for 3 s or when another behavior commences.
Preen Sitting	Moving the beak through feathers while sitting. Start at the first movement of beak moving through feathers. Ends when beak loses contact with feathers for 3 s or when another behavior commences.
Preen Standing	Moving the beak through the feather while standing. Start at the first movement of beak moving through feathers. Ends when beak loses contact with feathers for 3 s or when another behavior commences.

**Table 3:** BBWAP 6-point Gait Scoring System [35] modified from Kestin et al. [40].

Gait Score	Definition
0	The bird displays smooth, fluid locomotion. Typically, the foot is picked up and put down smoothly and each foot is brought under the bird’s center of gravity as it walks (rather than the bird swaying). Often, the toes are partially curled while the foot is in the air.
1	The bird has a slight defect in its gait that is difficult to define precisely. The bird may take unduly large strides, be unsteady, or wobble when it walks, which produces an uneven gait, but the problem leg is unclear/cannot be easily identified.
2	The bird has a definite and identifiable gait abnormality, but this does not affect its ability to move. The bird may make short, quick, unsteady steps with one leg, but is not sufficiently lame to seriously compromise its ability to move, i.e., maneuver, accelerate, and run.
3	The bird has an obvious gait defect that affects its ability to move. The bird may have a limp, jerky, or unsteady strut, or splay one leg as it moves. The bird often prefers to squat when not coerced to move and will not run.
4	The bird has a severe gait defect. The bird is capable of walking, but only with difficulty and when driven or strongly motivated. Otherwise, it squats down at the first available opportunity.
5	The bird is incapable of sustained walking on its feet. Although it may be able to stand, the bird cannot walk except with the assistance of the wings or by crawling on the shanks.

## Data Availability

The data presented in this study are available upon request from the corresponding author.

## Supplementary Materials

The following supporting information can be downloaded at https://www.mdpi.com/article/10.3390/ani13091432/s1, Table S1: Descriptive statistics demonstrating data distribution for each breed, Table S2: Descriptive statistics of last week mean activityA (%) and last week mean weight (kg) for each gait score and breed, Table S3: Generalized linear model results demonstrating the relationship between gait score and, the mean activityA (%) and mean weight (g). The means were calculated from data collected 8 days prior to bird gait being scored.
