# Supplementary material for: Individual Monitoring of Activity and Lameness in Conventional and Slower-Growing Breeds of Broiler Chickens Using Accelerometers
Source: Animals (Basel). 2023 Apr 22;13(9):1432. doi: 10.3390/ani13091432 (PMC10177109; doi:10.3390/ani13091432)
Supplement: Supplementary file 1 [file animals-13-01432-s001.zip › animals-2296593-supplementary.pdf]

animals

Supplementary Material

Individual Monitoring of Activity and Lameness in Conventional and Slower-Growing Breeds of Broiler Chickens Using Accelerometers

Justine Pearce *, Yu-Mei Chang and Siobhan Abeyesinghe

The Royal Veterinary College, Hawkshead Lane, North Mymms, Hatfield, AL9 7TA, UK
* Correspondence: justinepearce92@hotmail.co.uk

Table S1. Descriptive statistics demonstrating data distribution for each breed.

		CNV	SGH	SGN
Age in Days [1]	Median	27	41	40
	Minimum	26	26	26
	Maximum	30	49	48
	Mean ± SD	27.62 ± 1.60	38.49 ± 8.95	37.42 ± 9.22
Repeated Weight (kg) [2]	Median	1.44	1.68	1.61
	Minimum	1.11	0.62	0.64
	Maximum	1.91	2.74	2.75
	Mean ± SD	1.461 ± 0.227	1.563 ± 0.586	1.457 ± 0.587
Final Weight (kg) [3]	Median	2.32	2.08	2.41
	Minimum	1.84	1.75	1.89
	Maximum	2.78	2.74	2.88
	Mean ± SD	2.27 ± 0.30	2.13 ± 0.26	2.39 ± 0.29
ActivityA (%) [4]	Median	16.85	21.45	25.38
	Minimum	11.59	11.70	16.88
	Maximum	23.71	39.17	39.72
	Mean ± SD	17.61 ± 3.32	21.85 ± 6.12	26.57 ± 5.83
Gait Score	0	0	0	3
	1	1	17	10
	2	10	8	2
	3	4	0	0
Sex	Female	9	14	8
	Male	6	11	7

[1] Age at time of accelerometer attachment. [2] Weight recorded at each time of accelerometer attachment. [3] Weight recorded at welfare assessment. [4] ActivityA refers to the overall percent of time spent active measured using accelerometers. SD: standard deviation.

Table S2. Descriptive statistics of last week mean activityA (%) and last week mean weight (kg) for each gait score and breed.

	Gait Score	ActivityA (%)				Weight (kg)	Sample Size [1]
		Median	Min.	Max.	Mean ± SD	Mean ± SD	
SGH	**0**	-	-	-	-	-	0
	**1**	19.59	15.05	26.43	20.05 ± 3.02	1.83 ± 0.19	17
	**2**	16.86	13.90	23.74	17.70 ± 3.71	2.16 ± 0.15	8
	**3**	-	-	-	-	-	0
SGN	**0**	23.72	17.88	28.73	23.44 ± 5.43	1.57 ± 0.12	3
	**1**	24.19	17.99	28.44	23.65 ± 3.39	1.92 ± 0.29	10
	**2**	24.05	24.00	24.09	24.05 ± 0.06	2.07 ± 0.26	2

	**3**	-	-	-	-	-	0
CNV	**0**	-	-	-	-	-	0
	**1**	18.89	18.89	18.89	18.89	1.31	1
	**2**	17.19	11.59	20.44	16.89 ± 2.93	1.41 ± 0.12	10
	**3**	17.57	16.44	20.39	17.99 ± 1.69	1.59 ± 0.13	4
All Breeds	**0**	23.72	17.88	28.73	23.44 ± 5.43	1.57 ± 0.12	3
	**1**	20.60	15.05	28.44	21.29 ± 3.53	1.84 ± 0.25	28
	**2**	17.39	11.59	24.09	17.93 ± 3.70	1.77 ± 0.40	20
	**3**	17.57	16.44	20.39	17.99 ± 1.69	1.59 ± 0.13	4

[1] As a result of different breed hatching rates as part of the wider RSPCA BBWAP study [11] there were more pens of SGH birds compared to SGN and CNV birds. To keep sample sizes within pens consistent across breeds, accelerometers were attached to 15 SGN, 15 CNV and 25 SGH birds. SD: standard deviation

Table S3. Generalized linear model results demonstrating the relationship between gait score and, the mean activityA (%) and mean weight (g). The means were calculated from data collected 8 days prior to bird gait being scored.

		**OR**	**95% CI**		***p***
			**Lower**	**Upper**	
SGH	Mean Activity[A] (%)	0.81	0.53	1.25	0.348
	Mean Weight (g)	1.01	1.00	1.02	0.016
SGN	Mean Activity[A] (%)	1.05	0.76	1.44	0.770
	Mean Weight (g)	1.01	0.99	1.01	0.051
CNV	Mean Activity[A] (%)	1.11	0.66	1.85	0.689
	Mean Weight (g)	1.01	1.00	1.02	0.026

OR: Odds ratio >1 indicated a positive effect and odds ratio <1 indicated a negative effect of weight on behaviour; CI: Confidence Interval; *p*: *p* value
